# Portal Embolisation as Treatment of Severe Portal Hypertension Due to Idiopathic Intrahepatic Arterioportal Fistula: A Case Report

**DOI:** 10.1016/j.jceh.2023.10.006

**Published:** 2023-10-20

**Authors:** Anne J. Klompenhouwer, Adriaan Moelker, Sarwa D. Murad, Caroline M. den Hoed, Raoel Maan

**Affiliations:** ∗ Department of Gastroenterology and Hepatology, Erasmus MC University Medical Center, Rotterdam, The Netherlands; † Department of Radiology and Nuclear Medicine, Erasmus MC University Medical Center, Rotterdam, The Netherlands; ‡ The Erasmus MC Transplant Institute, Rotterdam, The Netherlands

**Keywords:** portal hypertension, intrahepatic arterioportal fistula, embolization

## Abstract

Intrahepatic arterioportal fistula (IAPF) is a rare cause of portal hypertension. Treatment is usually aimed at restoring the normal portal hemodynamics by obliterating the shunt. This report describes a case of idiopathic IAPF with severe portal hypertension complicated by portal enteropathy with vomiting, gastrointestinal hemorrhage and sepsis. The patient was successfully treated with portal embolization.

Intrahepatic arterioportal fistula (IAPF) is characterized by a vascular communication between the hepatic artery and portal vein. The condition can occur secondary to blunt or penetrating abdominal trauma, intrahepatic surgery, biopsy or other interventions. It may also be congenital, associated with Rendu-Osler-Weber or Ehlers Danlos syndrome or idiopathic.^1^ IAPF is a rare cause of portal hypertension and patients may present with complications of portal hypertension such as gastrointestinal bleeding, ascites, abdominal pain or discomfort and weight loss due to portal enteropathy with nausea and vomiting.^2^ The severity of symptoms is related to the volume of blood shunted thereby the portal pressure.

Treatment of IAPF is aimed at restoring the normal portal hemodynamics by obliterating the shunt, either endovascularly or surgically.^3^^,^^4^ In this report, we present a rare case of a patient with an acute presentation of a complex idiopathic IAPF with specific therapeutic challenges.

## Case presentation

**Figure 1:**
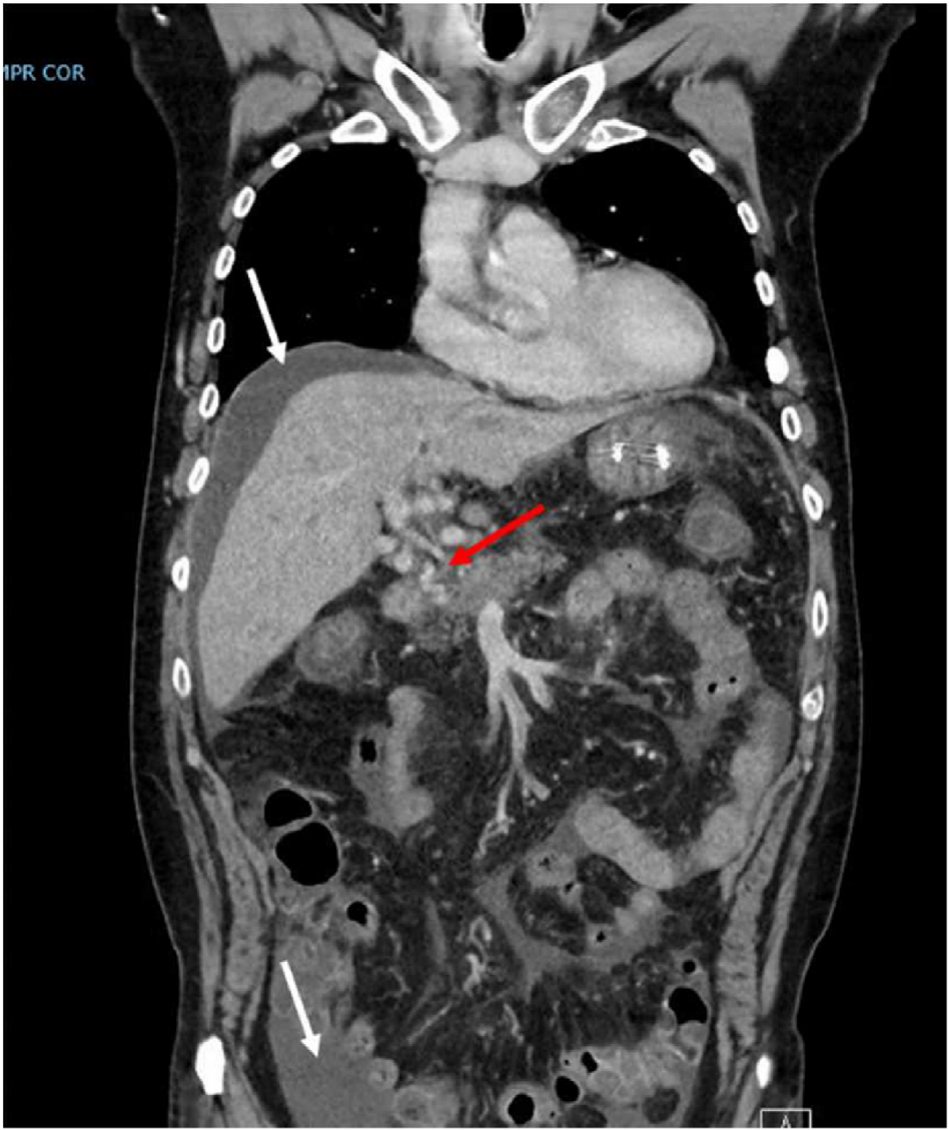
Coronal view of a CT scan at diagnosis showing portal hypertension with extensive collateral veins (red arrow) and ascites (white arrows).

A 66-year-old male with no medical history was referred to our tertiary referral center under the suspicion of hilar cholangiocarcinoma. He complained about recent onset abdominal pain, vomiting, weight loss and jaundice. Revision of the MRI and CT scan revealed arterioportal shunting due to IAPF from the left hepatic artery to the portal vein, with extensive collaterals (Figure 1). Additionally, it showed a thrombus in the left portal vein for which therapeutic anticoagulants were started, perihilar stenosis with biliary dilatation (left more than right) and ascites. There were no signs of liver cirrhosis, extensive laboratory investigations showed no signs of an inflammatory liver disease (antibodies were negative) and an additional PET-CT was made after which the suspicion of cholangiocarcinoma was rejected.

**Figure 2:**
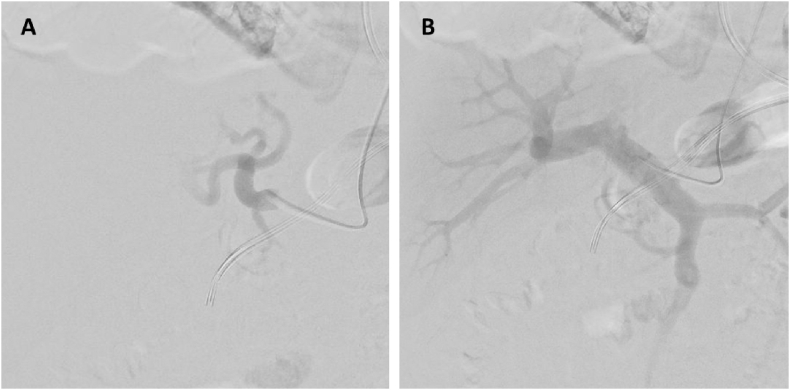
A: Arteriography of the hepatic artery. B: Diffuse network of fistulas between the portal vein and left hepatic artery with retrograde flow to the venous mesenterial system.

Patient was discussed in our vascular hepatology multidisciplinary meeting and was opted for endovascular embolization of the fistula. The procedure was approached transarterially (hepatic artery). During the procedure, the network of fistulas found between the portal vein and left hepatic artery was found to be so extensive that embolization of the complete fistula was not deemed possible (Figure 2). A second procedure was scheduled with a transhepatic portal approach in which a severely elevated portal pressure of 52 mmHg was measured. An attempt was made to embolize the fistula with coils upstream with an approach from the portal vein. This, however, did not result in a significant reduction in portal pressure or flow because of the complexity of connections between the hepatic artery and portal vein.

In the meantime, the patient clinically deteriorated with progressive weight loss and persistent vomiting due to portal enteropathy for which total parenteral nutrition was started. He developed a thrombosis in the mesenteric vein due to a low flow state, and a segmental thrombosis in the right PV. In addition, he showed signs of progressive portal hypertension, including gastrointestinal bleeding from esophageal varices and refractory ascites.

**Figure 3:**
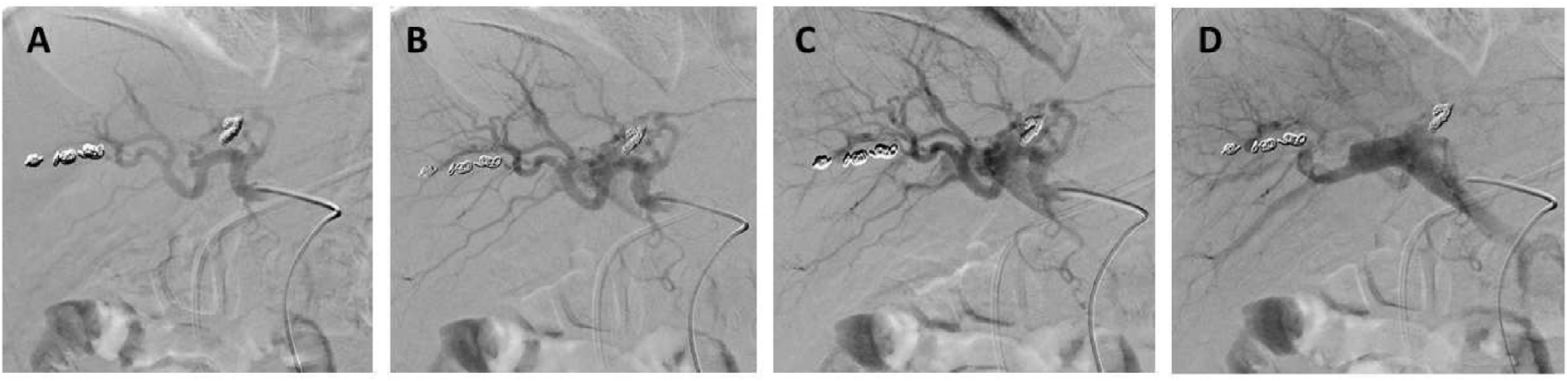
A: Arteriography of the hepatic artery. B: Extensive network of fistulas. C: Retrograde flow to the portal vein. D: Retrograde flow to the mesenterial veins. Note that the coils on the left side of each panel were placed before in an attempt to embolize the fistulas and to close the portal vein after the portogram.

**Figure 4:**
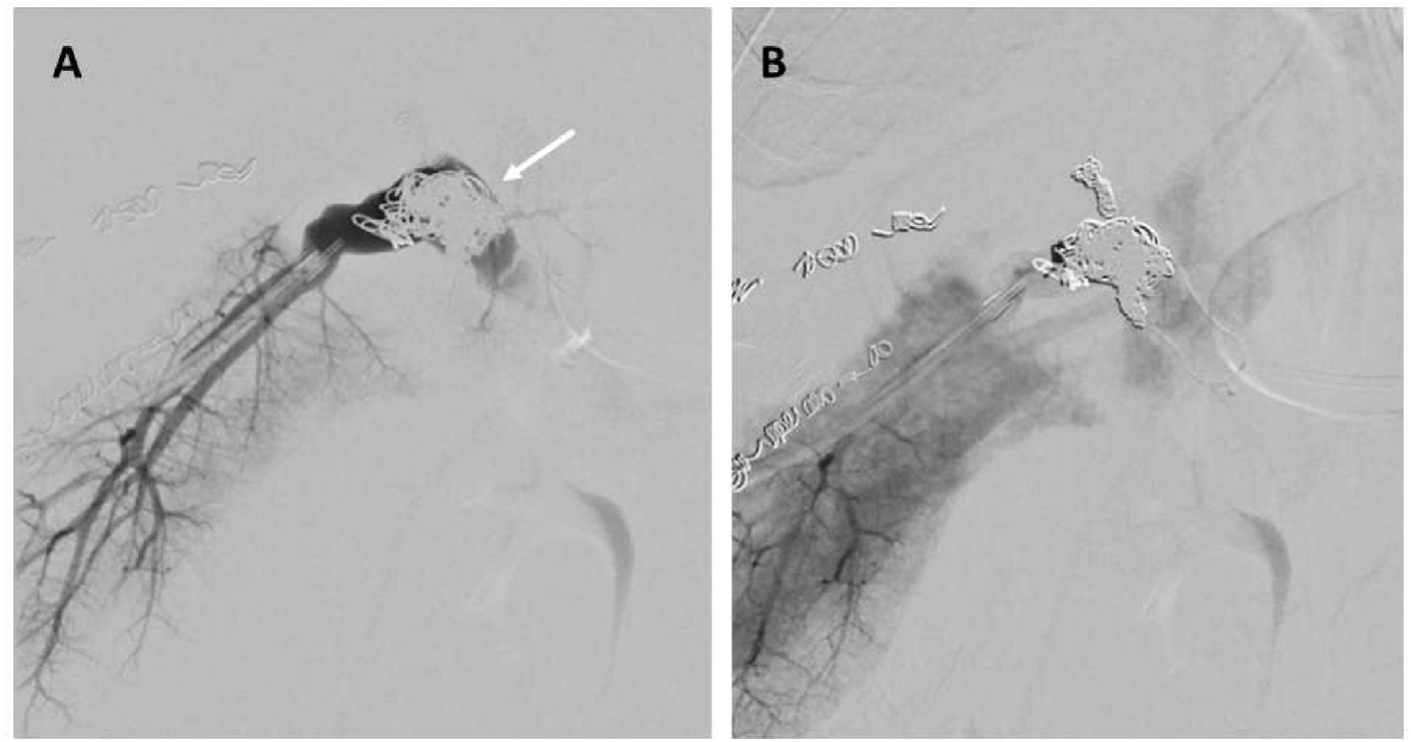
Portogram after portal vein embolization with Amplatzer plug (white arrow) and coils (A). Previously seen retrograde flow is absent (B).

We considered multiple treatment strategies. Firstly, we considered embolization of the proximal hepatic artery, which, given the portal vein thrombosis, would most likely result in a need for a left hemihepatectomy due to ischemia. During the subsequent angiography, the shunts, however, appeared to be even more extensive, as they were not limited to the left hepatic artery only, but involved the right hepatic artery as well (Figure 3). Therefore, this option was deemed impossible without immediate access to rescue liver transplantation. Secondly, primary liver transplantation was considered, however, given preserved liver function, his MELD score was low and a non-standard exception would need to be requested. Thirdly, we discussed the creation of a portosystemic shunt. The fistulae present close to the portal bifurcation would have made this technically very difficult, as the stent would have needed to enter the extrahepatic portal vein. More distal location to the left or right intrahepatic portal vein would make embolization of the portal vein branches and bifurcation impossible. In addition, the fistula would have been punctured when attempting to place a portosystemic shunt. Therefore, we decided against this option. Finally, we considered embolization of the main portal vein with the goal of pressure reduction in the mesenteric veins. As the left portal vein was completely obstructed and the right portal vein had segmental thrombosis, the intrahepatic portal flow was already suboptimal. As both the left and right hepatic arteries were intact, we hypothesized that this would give the liver sufficient perfusion after embolization of the main portal vein. The goal of portal vein embolization was to reduce the pressure in the mesenteric venous system leading to a diminution of the risk of bleeding. The portal hypertension was almost at arterial pressure due to the fistula. By portal vein occlusion, the mesenteric system lacked arterial pressure, reducing the risk of variceal bleeding. Additionally, the extensive network of mesenteric collaterals might be sufficient to ensure venous blood flow from the intestines to the liver. However, embolization of the main portal vein would make rescue liver transplantation surgically very challenging. While we were considering our options, the patient deteriorated even further and was admitted to the Intensive Care Unit (ICU) because of sepsis (*Escheria Coli*), most likely caused by bacterial translocation from the congested intestines. Due to this additional infectious complication, primary liver transplantation was contra-indicated, so our only option was complete portal embolization. The procedure was performed and the portal vein was embolized at the level of the hilus with an Amplatzer plug and packed with additional coils (Figure 4). This resulted in a direct reduction of the flow and the absence of retrograde flow into the mesenteric system.

**Figure 5:**
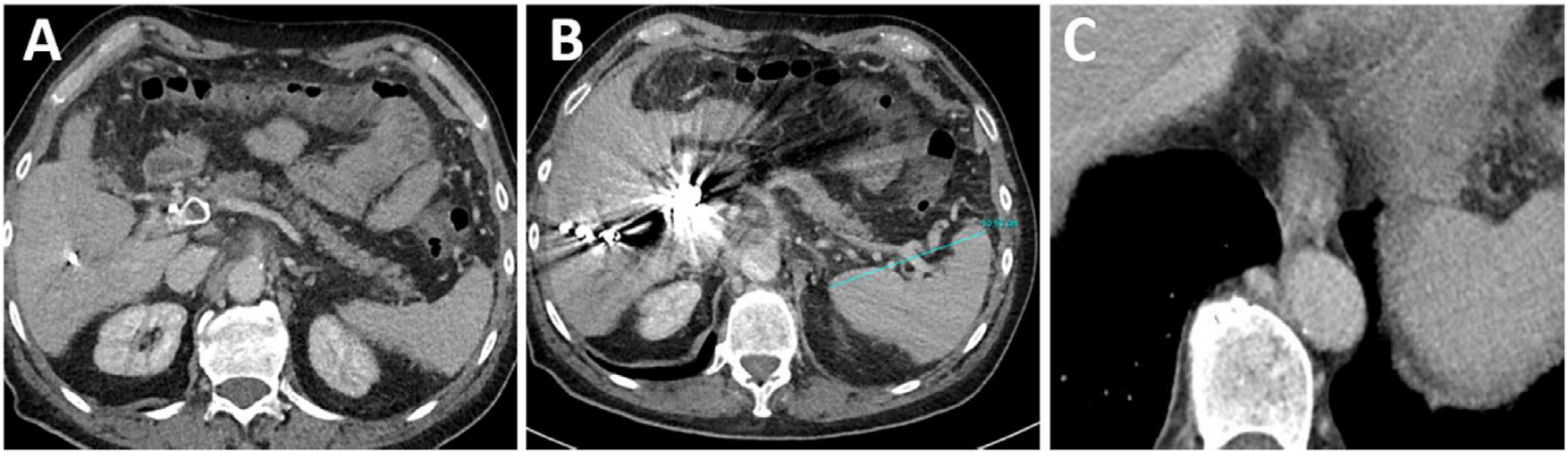
CT scan two years after the procedure. A: Intrahepatic coil and Aplatzer plug in het extrahepatic portal vein. Non-dilated splenic vein. B: Embolization materials in the liver and normal size of the spleen. C: Small esophageal varices and prominent azygos vein.

This procedure appeared successful. The sepsis could now be treated successfully and slowly oral nutrition could be restarted. He was discharged from the ICU after 24 days, and discharged home at hospital day 112. At home, he continued enteric tube feeds, oral anticoagulation, diuretics and non-selective beta-blockers. At follow-up three months after the portal vein embolization, tube feedings were discontinued and he was slowly gaining weight and physical stamina. After 12 months, he had normal weight and complete resolution of ascites and varices. The diuretics and non-selective beta-blockers could be discontinued. At that time, CT images also revealed the resolution of the mesenteric vein thrombosis. After 23 months, the CT scan revealed a non-dilated splenic vein, a normal size of the spleen in the small remaining esophageal varices (Figure 5).

The etiology of IAPF in this patient remains to date unclear. He has no clinical features of Rendu-Osler-Weber or Ehlers Danlos syndrome.

## Discussion

When IAPF causes symptomatic portal hypertension, interventional treatment is required.^5^ Traditionally, surgical treatment was performed by either ligation of the hepatic artery supplying the IAPF or resection of the vascular anomaly itself.^6^ Over the past decades however, endovascular techniques have improved and have shown to have lower morbidity and mortality as compared to surgical treatment.^5^^,^^7^

The endovascular technique usually entails embolization of the proximal site of the fistula branches.^8^ When this is insufficient or the fistulas are extensive, embolization of the supplying hepatic artery branches can be successful in reducing the flow.^7^ In this case however, there was intrahepatic portal vein thrombosis and the fistulas were so extensive and supplied by both the right as the left hepatic artery, that embolization of the feeding fistula or supplying hepatic artery branches was deemed impossible. After careful consideration and as the patient had extensive mesenterial collateral veins, we performed an embolization of the portal vein. This resulted in a direct reduction of the flow and after twelve months of follow-up, no signs of portal hypertension were present. To our knowledge, this is the first case report describing portal embolization as a treatment of IAPF.

IAPF is a rare cause of portal hypertension that may cause serious complications, depending on the volume of blood shunting. Treatment is aimed at restoring the normal portal hemodynamics by obliterating the shunt. When there is intrahepatic portal vein thrombosis and the fistulas are so extensive that embolization is deemed impossible, portal embolization may be considered as a treatment when extensive collateral veins are present.

## Credit authorship contribution statement

AJK & RM: conceptualization and writing (original draft).

SDW & CMdH: writing (review & editing).

AM: figures and writing (review & editing).

## Conflicts of interest

None.

## Funding

None.
